# Mapping an epitope in EBNA‐1 that is recognized by monoclonal antibodies to EBNA‐1 that cross‐react with dsDNA

**DOI:** 10.1002/iid3.119

**Published:** 2016-08-02

**Authors:** Pragya Yadav, Matthew T. Carr, Ruby Yu, Alice Mumbey‐Wafula, Linda A. Spatz

**Affiliations:** ^1^ Department of ChemistryCity College of New York160 Convent AvenueNew YorkNew York10031; ^2^ Graduate Program in BiochemistryGraduate Center of the City University of New York160 Convent AvenueNew YorkNew York10031; ^3^ Department of BiologyCity College of New York160 Convent AvenueNew YorkNew York10031; ^4^ Department of Pathobiology, Sophie Davis School of Biomedical EducationCity College of New York160 Convent AvenueNew YorkNew York10031

**Keywords:** anti‐dsDNA antibodies, autoantibody, cross‐reactivity, EBNA‐1, molecular mimicry, SLE

## Abstract

**Introduction:**

The Epstein Barr Virus (EBV) has been associated with the autoimmune disease, Systemic Lupus Erythematosus (SLE). EBV nuclear antigen‐I (EBNA‐1) is the major nuclear protein of EBV. We previously generated an IgG monoclonal antibody (MAb) to EBNA‐1, 3D4, and demonstrated that it cross‐reacts with double stranded DNA (dsDNA) and binds the 148 amino acid viral binding site (VBS) in the carboxyl region of EBNA‐1. The aim of the present study was to characterize another antibody to EBNA‐1 that cross‐reacts with dsDNA, compare its immunoglobulin genes to 3D4, and finely map the epitope in EBNA‐1 that is recognized by these cross‐reactive antibodies.

**Methods:**

We generated an IgM MAb to EBNA‐1, 16D2, from EBNA‐1 injected mice and demonstrated by ELISA that it cross‐reacts with dsDNA and binds the 148 amino acid VBS. We sequenced the variable heavy and light chain genes of 3D4 and 16D2 and compared V gene usage. To more finely map the epitope in EBNA‐1 recognized by these MAbs, we examined their binding by ELISA to 15 overlapping peptides spanning the 148 amino acid domain.

**Results:**

Sequence analysis revealed that 3D4 and 16D2 utilize different V_H_ and V_L_ genes but identical J_H_ and J_k_ regions with minimal junctional diversity. This accounts for similarities in their CDR3 regions and may explain their similar dual binding specificity. Epitope mapping revealed 3D4 and 16D2 bind the same peptide in the VBS. Based on the crystal structure of EBNA‐1, we observed that this peptide resides at the base of an exposed proline rich loop in EBNA‐1.

**Conclusion:**

We have demonstrated that two MAbs that bind EBNA‐1 and cross‐react with dsDNA, recognize the same peptide in the VBS. This peptide may serve as a mimetope for dsDNA and may be of diagnostic and therapeutic value in SLE.

## Introduction

Autoimmune diseases such as Systemic Lupus Erythematosus (SLE) are caused by a constellation of genetic, environmental, and hormonal factors. Environmental triggers include pathogens that may elicit an autoimmune response as a consequence of molecular mimicry. Some viruses that have been associated with SLE are herpes zoster, cytomegalovirus (CMV), human endogenous retroviruses and the Epstein Barr Virus (EBV) 1, 2, 3, 4, 5. Specific proteins and peptides derived from these viruses are thought to mimic nuclear self antigens and thereby elicit antibodies that cross‐react with these autoantigens.

The association of EBV with SLE originally stems from observations that the prevalence of EBV infection is higher in SLE adolescents than age matched controls, that EBV infection sometimes precedes SLE, and that EBV DNA and EBV viral load are higher in SLE patients than healthy individuals 2, 6, 7, 8, 9, 10. In addition, SLE patients have elevated expression of EBV mRNA's and a higher frequency of antibodies to EBV early antigens, capsid antigens, and latent membrane proteins than controls 11, 12, 13, 14. Further evidence linking EBV to SLE is based on studies demonstrating that antibodies to the Epstein Barr virus nuclear antigen 1 (EBNA‐1), the major nuclear protein in EBV which is expressed in all EBV infected cells, cross‐react with the splicesomal ribonucleoproteins, Sm B/B′, Sm D, nRNPs, and Ro. Antibodies to Sm are observed in approximately 25% and antibodies to Ro in approximately 50% of lupus patients 2, 15. Sabbatini *et al*. 16 observed that a G‐R repeat region in Sm D is homologous to a region in EBNA‐1 and demonstrated that SLE sera that recognized a G‐R peptide derived from Sm D, also recognized the G‐R peptide derived from EBNA‐1. Mice immunized with this EBNA‐1 peptide, developed antibodies to Sm D as well as to the immunizing peptide.

James and Harley 17 identified a proline rich peptide in EBNA‐1 (PPPGRRP) that is homologous to a proline rich peptide in Sm B/B′ (PPPGMRPP). They showed that sera from lupus patients but not controls bound the EBNA‐1 derived peptide and that rabbits and mice immunized with PPPGRRP from EBNA‐1 developed antibodies that cross‐reacted with Sm B/B′ as well as nRNPs 18, 19. In a subsequent study, McClain *et al*. 20 observed that another peptide in EBNA‐1 elicited a cross‐reactive response to a peptide in the ribonucleoprotein, Ro. Interestingly, no primary sequence homology was observed between the EBNA‐1 and Ro peptides but both were noted to have similarly high isoelectric points and to share structural similarities. Together these studies reveal how a viral antigen can act as a molecular mimic for an autoantigen and illustrate the role of molecular mimicry in the etiology of lupus autoantibodies.

Antibodies to double stranded DNA (dsDNA) are the hallmark of SLE and are found in 50–80% of SLE patients. Although dsDNA is a target of anti‐dsDNA antibodies, it is not known whether dsDNA is the eliciting antigen. Screening of phage peptide libraries has identified peptides derived from autoantigens that serve as molecular mimics for dsDNA 21, 22, 23. Antibodies to nRNPs have been shown to cross‐react with dsDNA 24. Studies in the laboratory of John Harley have shown that some animals immunized with PPPGRPP developed antibodies to dsDNA, but they hypothesized that this was due to epitope spreading rather than cross‐reactivity 18, 19, 20.

We previously demonstrated that mice injected with an EBNA‐1 expression vector or the EBNA‐1 protein, developed antibodies to dsDNA 25, 26. To determine the basis of this anti‐dsDNA response, we generated IgG monoclonal antibodies (MAbs) to EBNA‐1 from EBNA‐1 injected mice and tested them for cross‐reactivity with dsDNA. We identified an IgG MAb designated 3D4 and demonstrated that it cross‐reacts with dsDNA. We showed that this MAb recognizes a 148 amino acid domain in the carboxyl region of EBNA‐1 25.

We now describe a recently generated IgM MAb to EBNA‐1 (16D2) that also cross‐reacts with dsDNA. Although this MAb utilizes different heavy and light chain genes than 3D4, it recognizes the same carboxyl domain in EBNA‐1. The present study was undertaken to more finely map the peptide epitope in EBNA‐1 that can serve as a peptide mimic for dsDNA. By screening an overlapping peptide library derived from this 148 amino acid domain, we have identified a peptide target of both 3D4 and 16D2. Understanding how a peptide derived from a pathogen can serve as a molecular mimic for dsDNA may have important implications for diagnostic and therapeutic strategies in SLE.

## Materials and Methods

### Purification of rEBNA‐1 lacking the Gly‐Ala repeat

Recombinant EBNA‐1 (rEBNA‐1) lacking most of the G‐A repeat was purified from a baculovirus expression vector kindly provided by Dr. Lori Frappier (University of Toronto, Toronto, Canada), as previously described 25, 27. The rEBNA‐1 protein contains a 6x His tag at the amino termini, which allowed for its isolation on Ni^2+^‐NTA beads.

### Mouse immunization and somatic cell fusion

BALB/c mice were injected intraperitoneally with 50 μg of rEBNA‐1 in complete Freund's adjuvant (CFA) 1:1(v/v) and boosted at weeks 3, 7, and 12 with 25 μg of rEBNA‐1 in incomplete Freund's adjuvant (IFA) 1:1(v/v). Mice were serially bled and sera examined by ELISA for antibodies to EBNA‐1 and dsDNA. Three days after the last boost, mouse splenocytes were harvested and fused to NSO cells according to Iliev *et al*. 28. Hybridoma, supernatants were tested by ELISA for IgM and IgG antibodies to EBNA‐1 and dsDNA. Hybridomas producing MAbs to EBNA‐1 that cross‐react with dsDNA or non dsDNA binding anti‐EBNA‐1 MAbs were subcloned two times at limiting dilution. The MAbs were grown in serum free media and IgG MAbs were purified on protein G sepharose columns.

### Isolation of truncated recombinant EBNA‐1 proteins

PCR fragments encoding the amino and carboxyl regions of rEBNA‐1 were previously cloned into a pET28a *Escherichia coli* expression vector carrying an N‐terminal His tag, as previously described 25. The amino fragment (LS8) encompassing aa 1–404 and lacking the G‐A repeat and the carboxyl fragment (LS9) encompassing aa 410–641 were isolated from IPTG induced *E. coli* lysates and purified on Ni^2+^‐NTA beads according to Yadav *et al*. 25.

Plasmids (pET15b) carrying an N‐terminal His tag and expressing the following truncated EBNA‐1 proteins; EBNA_452‐641_, EBNA_459‐619,_ EBNA_459‐607,_ were a generous gift from Dr. Lori Frappier 29. These fragments were expressed in *E. coli* strain BL21 (DE3) and isolated from cell‐lysates on Ni^2+^‐NTA beads 25.

### Crithidia luciliae assay

Ready to use Crithidia slides (Antibodies Inc., Davis, CA) were immunostained with MAb, 16D2 at 10 μg/ml. Slides were incubated in a moist chamber for 30 min at room temperature, washed extensively in PBS and then immunostained with a 1:1000 dilution of goat anti‐mouse IgM FITC (Southern Biotech, Birmingham, AL) for 30 min at room temperature. Slides were washed again in PBS and then were examined by fluorescence microscopy using a Nikon Eclipse microscope, TE 2000‐S at 40× magnification.

### Ig heavy and light chain cDNA synthesis and sequencing

Total RNA was isolated from hybridomas producing MAbs 3D4 or 16D2, using Trizol reagent (Life Technologies, Thermo Fisher Scientific, Waltham, MA) according to the manufacturers protocol. First strand cDNA was prepared by RT‐PCR using 3.5 μg of RNA, 50 ng of random hexamers and 200U of superscript III RT (Invitrogen, Life Technology, Carlsbad, CA, USA) according to the manufacturers protocol.

Two rounds of PCR amplification were performed for the Ig heavy and light chain genes using 2.0 μl cDNA (for the first PCR) or 3.0 μl of first PCR product (for the second PCR), 250 μM of each dNTP, 0.5 μM of each primer, and 1.5 U of Hot Star Taq polymerase (Qiagen, Germantown, MD) in a total reaction mixture of 50 μl. The first round of PCR was performed at 94°C for 15 min followed by 50 cycles of 94°C for 30 sec, 56°C (IgH) or 50°C (Igκ) for 30 sec, 72°C for 55 sec, and a final extension at 72°C for 10 min according to Tiller *et al*. 30. Primers used for the first PCR were 5′mVHE (GGGAATTCGAGGTGCAGCTGCAGGAGTCTGG) and 3′Cγ1 outer (GGAAGGTGT GCACACCGCTGGAC) for the γ1 heavy chain gene, 5′mVHE and 3′Cμ outer (AGGGGGCTCTCGCAGGAGACGAGG) for the μ heavy chain gene, and 5′ mVk (GAYATTGTGMTSACMCARWCTMCA) and 3′ Ck outer (ACTGAGGCACCT CCAGATGTT) for the kappa light chain gene. Semi‐nested second round PCR was performed at 94°C for 15 min, followed by 50 cycles of 94°C for 30 sec, 60°C (for IgH) or 45°C (for Igκ) for 30 sec, 72°C for 45 sec and a final extension at 72°C for 10 min 30. Primers used for the second PCR were 5′ mVHE and 3′Cγ1 inner (GCTCAGGGAAATAGGCCTTGAC) for the γ1 heavy chain, 5′ mVHE and 3′Cμ inner (AGGGGGAAGACATTTGGGAAGGAC) for the μ heavy chain genes and 5′mVκ and 3′ Cκ inner (TGGGAAGATGGATACAGTT) for the kappa light chain gene (Christian Busse, personal communication). PCR products from the 2nd PCR were analyzed on a 2% agarose gel and sequenced (Macrogen, Rockville, MD). Germline heavy and light chain genes were identified by IgBLAST.

### ELISAs

#### Direct ELISAs for binding to dsDNA, EBNA‐1, LPS, Sm, and Proteinase 3

ELISAs to examine direct antibody binding to EBNA‐1, dsDNA, truncated fragments of EBNA‐1, LPS, Sm, and Proteinase 3 were as previously described 25, 26, 31. Either goat anti‐mouse IgM or goat anti‐mouse IgG conjugated to alkaline phosphatase (AP) (Southern Biotech) were used as secondary antibodies to detect binding of 16D2 or 3D4, respectively, to antigen coated plates. Isotype ELISAs to determine whether additional IgG MAbs were of the IgG1, IgG2a, IgG2b, or IgG3 subclass were performed as previously described 25.

#### Anti‐peptide ELISAs

Fifteen peptides containing a five amino acid overlap, derived from a 148 amino acid region in the viral dimerization/binding domain in the carboxyl region of EBNA, were synthesized and biotinylated at the amino termini by LifeTein LLC (Hillsborough, NJ). Purity of all peptides was greater than 80%. Costar ELISA plates (Corning Inc., Corning, NY) were coated overnight at 4°C with a 1:1000 dilution of streptavidin in PBS (Southern Biotech). The next day, plates were blocked in PBS, 1% BSA, for 2 h at room temperature. Plates were then incubated with 100 μg/ml of biotinylated‐peptide (50 μl per well) for 1 h at 37°C followed by six washes in PBS, 0.05% tween 20. Plates were next incubated for 1 h at 37°C with 5 μg/ml of 3D4 or 10 μg/ml of 16D2 diluted in PBS, 0.1% BSA. Plates were washed and then incubated with a 1:1000 dilution of goat anti‐mouse IgG‐AP or goat anti‐mouse IgM‐AP to detect 3D4 or 16D2 binding, respectively. Plates were developed with 4‐nitrophenyl phosphate disodium hexahydrate as substrate and read at 405 nm on a Titertek Multiscan ELISA reader. To compare the binding of 16D2 or 3D4 to biotinylated EBNA‐1 versus biotinylated PFM‐15 peptide, ELISAs were performed as described above except that following the coating with streptavidin, plates were incubated with mole equivalents of biotinylated PFM‐15 or biotinylated EBNA‐1. EBNA‐1 was biotinylated using EZ‐Link Sulfo‐NHS‐Biotinylation Kit (Thermo Scientific, Rockford IL). Following biotinylation, biotinylated EBNA‐1 (B‐EBNA‐1) was applied to a Zeba Desalt Spin Column (Thermo Scientific) and then dialyzed against PBS to remove excess biotin.

#### Peptide inhibition ELISAs

For peptide inhibition of MAb binding to dsDNA, 3D4 (2.5 μg/ml) and 16D2 (5 μg/ml) were pre‐incubated for 1 h at 37°C with increasing concentrations of peptide PFM‐15 or GC‐15 (concentrations of peptide ranging from 0.1 μg/ml to 100 μg/ml) and then transferred to pre‐blocked dsDNA coated Immulon‐2 plates (Dynatech Laboratories Inc., Chantilly, VA). Plates were then incubated for 2 h at 37°C, washed, and then incubated with 1:1000 dilution of goat anti‐mouse IgG‐AP or goat anti‐mouse IgM‐AP to detect 3D4 or 16D2 binding, respectively. Plates were developed with substrate as described above. MAbs were used at a concentration resulting in 50% maximal binding to dsDNA.

For peptide inhibition of MAb binding to EBNA‐1, 3D4 (0.05 μg/ml) and 16D2 (5 μg/ml) were incubated with increasing concentrations of peptide PFM‐15 or GC‐15, ranging from 0.1 μg/ml to 1 mg/ml and then transferred to pre‐blocked EBNA‐1 coated Costar plates. Plates were then incubated for 1 h at 37°C, and the remainder of the ELISA was performed as described above. MAbs were used at a concentration resulting in 50% optimal binding to EBNA‐1.

## Results

**Figure 1:**
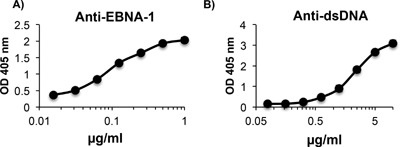
IgG antibody to EBNA‐1 cross‐reacts with dsDNA. IgG MAb, 3D4 binds strongly to EBNA‐1 (A) and cross‐reacts with dsDNA (B) as determined by ELISA. ELISAs are representative of three experiments. ELISA values were obtained in triplicate. Error bars indicate standard deviations of triplicates.

We previously demonstrated that some mice immunized with EBNA‐1 developed antibodies to EBNA‐1 that cross‐reacted with dsDNA. We generated anti‐EBNA‐1 MAbs from one of these mice and observed that 71% of the anti‐EBNA‐1 MAbs cross‐reacted with dsDNA 25. Pragya *et al*. 25 described an IgG MAb, designated 3D4, that binds with high affinity to EBNA‐1 and cross‐reacts with dsDNA (Fig. 1A and B). Epitope mapping revealed that 3D4 recognizes a 148 amino acid fragment in the carboxyl region of EBNA‐1 25.

### Characterization of an IgM anti‐EBNA‐1 MAb (16D2) that cross‐reacts with dsDNA

**Figure 2:**
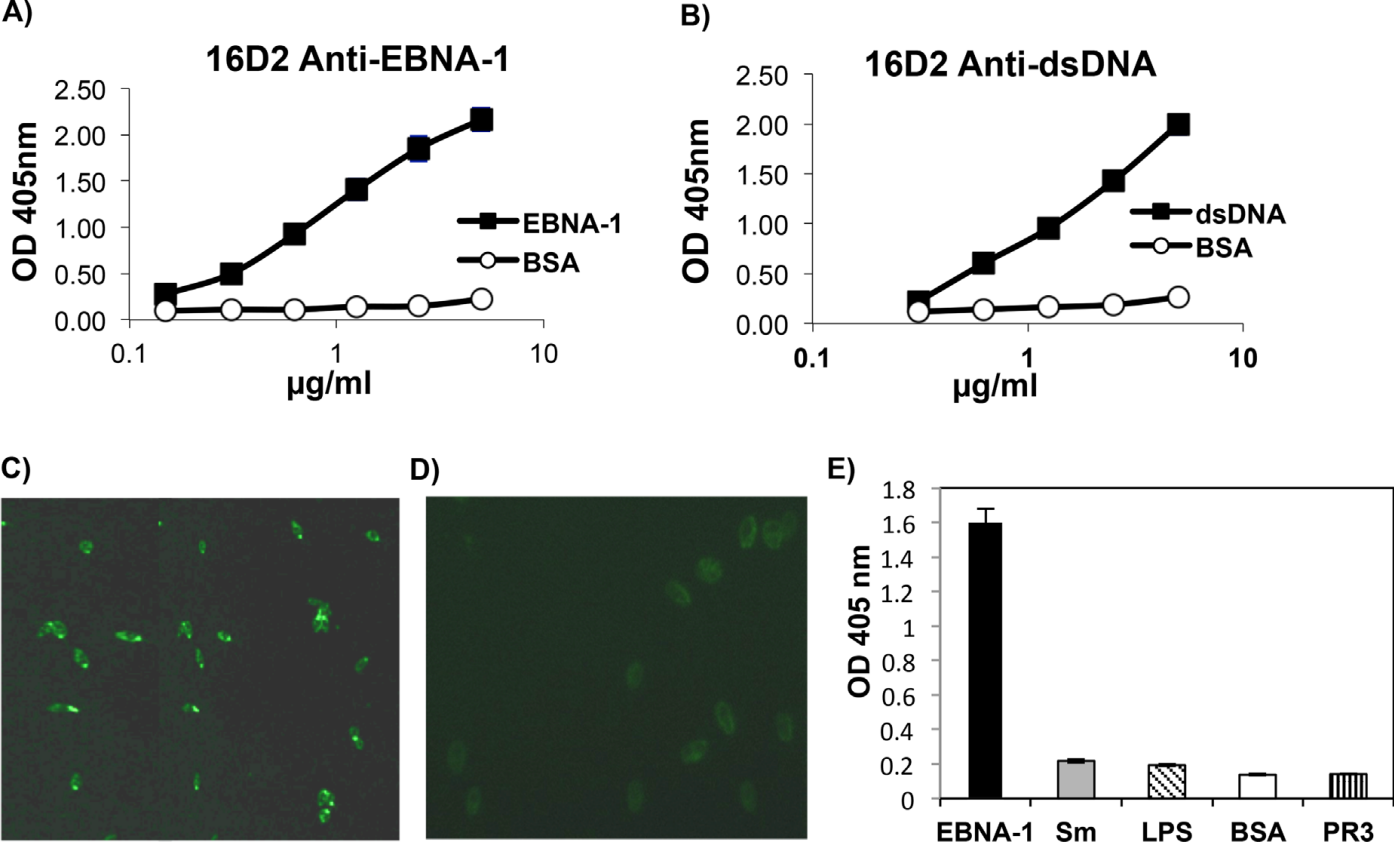
16D2 cross‐reacts with dsDNA. 16D2 was tested by ELISA for binding to EBNA‐1 (A) and dsDNA (B). Binding to dsDNA was confirmed by immunostaining of the kinetoplast of Crithidia luciliae (C). Immunostaining of an isotype control MAb is shown in D (D). 16D2 was tested by ELISA for reactivity with a panel of antigens; Sm, LPS, BSA, and proteinase 3 (PR3) (E). Results are representative of three experiments. ELISA values were obtained in triplicate. Error bars indicate standard deviations of triplicate values.

In the present study, we characterized an IgM MAb to EBNA‐1, designated 16D2, that was generated from somatic cell fusion following injection of a mouse with recombinant EBNA‐1 protein lacking the glycine alanine repeat region, as previously described 25. We demonstrated by ELISA that 16D2 binds EBNA‐1 and cross‐reacts with dsDNA, (Fig. 2A and B). Further confirmation of cross‐reactivity with dsDNA was demonstrated by binding of 16D2 to the kinetoplast, a dsDNA containing organelle, of the protozoa, Crithidia luciliae (Fig. 2C). Since IgM antibodies are part of an early immune response and are often polyreactive, we examined 16D2 for its polyreactivity. 16D2 was tested by ELISA for binding to a panel of antigens frequently recognized by polyreactive antibodies (Fig. 2E). 16D2 was not observed to be polyreactive as it did not bind BSA, LPS, Sm, or Proteinase 3 (PR3), a target autoantigen in Wegener's Granulomatosis.

### 16D2 recognizes an epitope in the carboxyl region of EBNA‐1

**Figure 3:**
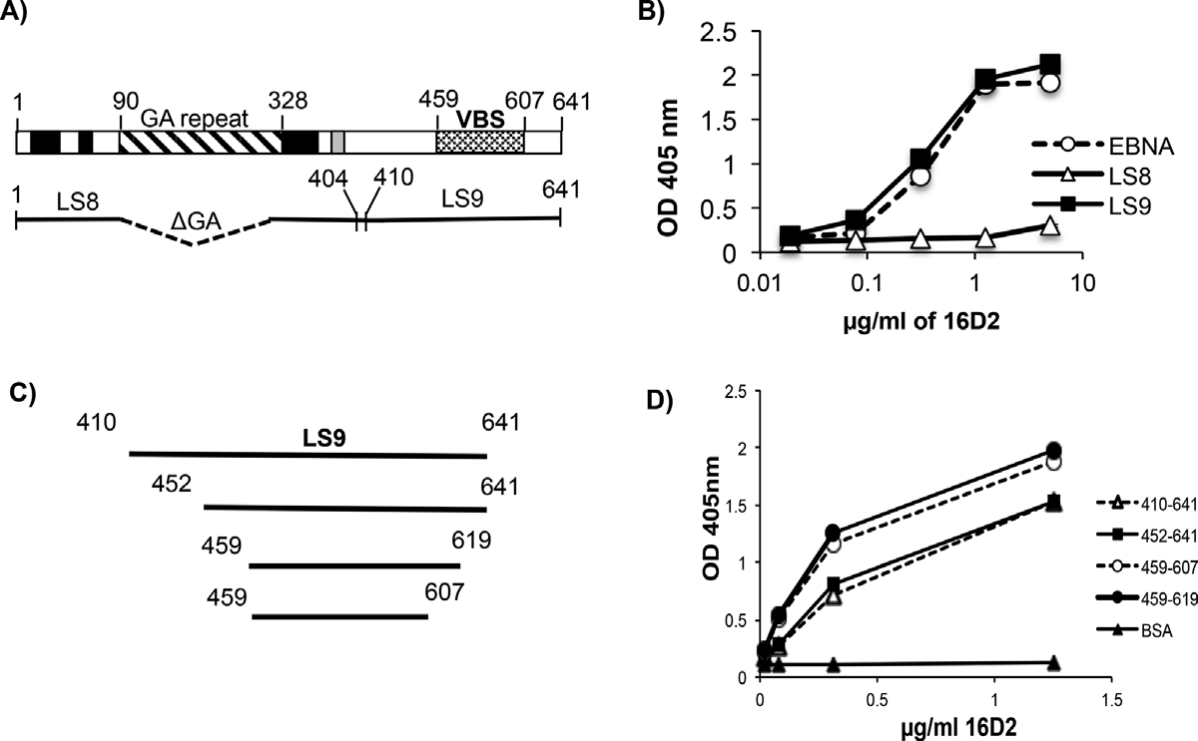
16D2 binds to an epitope in the carboxyl region of EBNA‐1. (A) Open reading frame map of EBNA‐1 showing the amino (LS8) and carboxyl (LS9) region. (B) 16D2 was tested by ELISA for binding to LS8 and LS9 and shown to bind LS9 only. (C) Schematic of three truncated recombinant fragments of LS9 expressed in and purified from *E. coli*. (D) 16D2 was tested by ELISA for binding to the truncated fragments, aa 452–641, aa 459–619, and aa 459–607 and compared to the binding to LS9 (aa 410–641). ELISAs are representative of two experiments. ELISA values were obtained in triplicate. Error bars indicate standard deviations of triplicates.

Since 16D2, was observed to bind EBNA‐1 and cross‐react with dsDNA, we wondered whether it would recognize the same epitope in EBNA‐1 as 3D4. We therefore examined 16D2 for reactivity with the amino and/or carboxyl regions of EBNA‐1. Recombinant EBNA‐1 proteins representing the amino and carboxyl regions of EBNA‐1 were purified in our laboratory from *E. coli* plasmid expression vectors as described by Yadav *et al*. 25 and were coated on ELISA plates. The amino fragment designated LS8 contains amino acids 1–404 (lacking the G‐A repeat), whereas, the carboxyl fragment designated LS9 contains amino acids 410–641 (Fig. 3A). 3D4 was previously shown to bind LS9 but not LS8. In the present study, we demonstrated that 16D2 also binds LS9 but not LS8 (Fig. 3B). We next tested 16D2 for binding to three truncated recombinant protein fragments derived from the carboxyl region of EBNA‐1; EBNA_452‐641_, EBNA_459‐619_, and EBNA_459‐607_ (Fig. 3C). These fragments were purified from recombinant *E. coli* expression plasmids kindly provided to us by Dr. Lori Frapier (University of Toronto). 16D2 was shown to bind strongly to all 3 fragments (Fig. 3D). The smallest fragment, which is 148 amino acids (aa) long (EBNA_459‐607_), corresponds to the viral dimerization/DNA binding site (VBS) of EBNA‐1. 3D4 was previously shown to bind strongly to this fragment as well 25.

### Comparison of the relative binding of 3D4 and 16D2 to EBNA‐1

**Figure 4:**
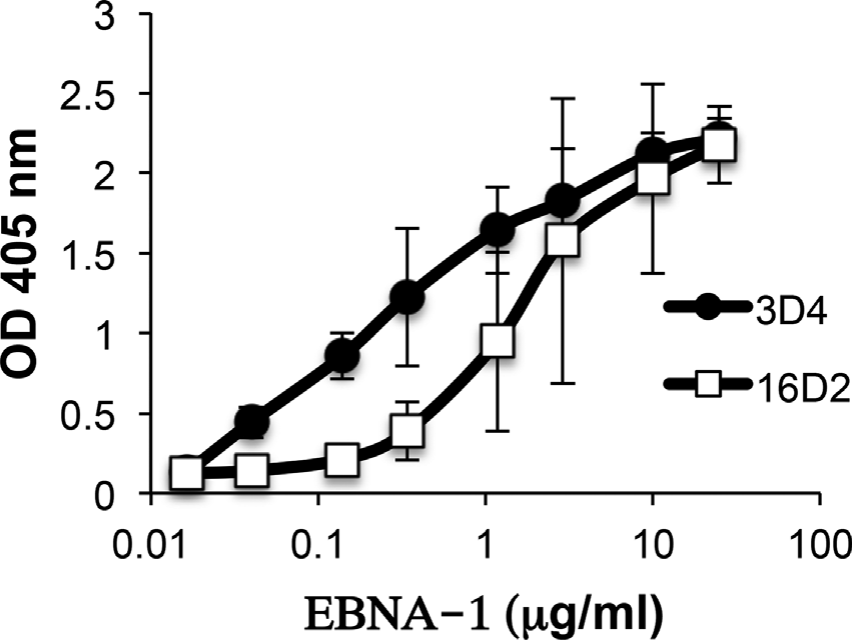
Comparison of the binding of 3D4 and 16D2 to EBNA‐1. The binding of 16D2 and 3D4 to EBNA‐1were compared by ELISA. Goat anti‐mouse kappa‐AP was used as secondary antibody to detect the binding of 16D2 (an IgM MAb) and 3D4 (an IgG MAb) in the same assay. Results represent the average of two experiments. Standard deviation is indicated by error bars.

Comparison of the relative binding of 16D2 and 3D4 to EBNA‐1 was demonstrated by ELISA. Both antibodies were serially diluted and incubated on the same EBNA‐1 coated ELISA plate and goat anti‐mouse kappa‐AP was added as secondary antibody (Fig. 4). In two out of two trials, the binding of 3D4 to EBNA‐1, as measured by OD values was relatively higher than the binding of 16D2 to EBNA‐1, within the range of 0.04–1 μg/ml of antibody. This suggests, but does not prove that 3D4 binds more strongly to EBNA‐1 than 16D2. However, other factors can account for these observations and accurate measurement of antibody binding strength and affinity will require precise kinetic binding assays such as surface plasmon resonance.

### Sequence analysis of the V_H_ and V_L_ genes of 3D4 and 16D2

The variable heavy (V_H_) and light chain (V_L_) genes of 3D4 and 16D2 were sequenced to determine their identity. Sequences were analyzed using Ig BLAST (NCBI database) to determine the closest germline genes of origin. Both antibodies were found to utilize different V_H_ and V_L_ genes but the same J_H_ and J_k_ gene segments (Tables 1 and 2). 3D4 was found to have 99% homology to the V_H_ germline gene VH7183.14 and 98.3% homology to the V_L_ germline gene, bb1. 16D2 was found to have the closest homology (95.8%) to the germline gene, V_H_ J558.16 and 100% homology to the germline V_L_ gene, 8–30. 3D4 has three mutations in its V_H_ region (Supplementary Fig. S1) and five mutations in its V_L_ region (Supplementary Fig. S2). 16D2 has 12 mutations in its V_H_ region, six of which are silent (Supplementary Fig. S3). The 16D2V_L_ is identical to the germline gene 8–30 (Supplementary Fig. S4). Interestingly, 16D2 and 3D4 utilize the same J_H_2 segment in their V_H_ regions and the same J_K_1 in their V_L_ regions. Furthermore, the D‐J joints of both MAbs lack junctional diversity as the carboxy terminal YFDYW amino acids of both CDR3s are J_H_2 germline encoded (Table 1). The carboxy terminal PWTF amino acids in the V_k_ CDR3s of 3D4 and 16D2 are both J_k_2 germline encoded. 16D2 was noted to contain three positively charged arginine residues in its CDR3V_H_ region, while 3D4 has one. Arginine residues are frequently observed among anti‐dsDNA antibodies 32, 33. The lengths of the V_H_ CDR3 regions in 3D4 and 16D2 (33 and 39 amino acids, respectively) are slightly longer than normal a characteristic of many autoreactive and polyreactive antibodies 34, 35. The lengths of the V_L_ CDR3 regions of 3D4 and 16D2 (30 amino acids) are average. The length of the CDR3 regions were determined by counting the amino acid residues after the conserved cysteine at the end of FR3 to the conserved tryptophan‐glycine in all mouse J_H_ segments or the conserved phenylalanine‐glycine in all J_κ_ segments 30.

**Table 1:** V_H_ genefor correctness.</query>‐‐> analysis of 3D4 and 16D2

Heavy Chain							
MAb	Class	GermlineV_H_	D	J_H_	CDR3 length	CDR3 aa	# mut.
3D4	γ	VH7183.14	NA	J_H_2	33	ARPPPFYFDYW	3
16D2	μ	J558.16	D1‐2*01	J_H_2	39	ARILRLRQYFDYW	12

NA, not available. Stretches of germline encoded amino acid (aa) homology are underlined. # mut; number of mutations.

**Table 2:** V_L_ gene analysis of 3D4 and 16D2

Light Chain						
MAb	Class	GermlineV_K_	J_K_	CDR3 length	CDR3 aa	# mut.
3D4	κ	bb1	J_K_1	30	SQTTHVPWTF	5
16D2	κ	8‐30	J_K_1	30	QQYYSYPWTF	0

Germline encoded stretches of amino acid (aa) homology are underlined. # mut; number of mutations.

### Mapping the peptide epitope in EBNA‐1 that is recognized by 3D4 and 16D2

**Figure 5:**
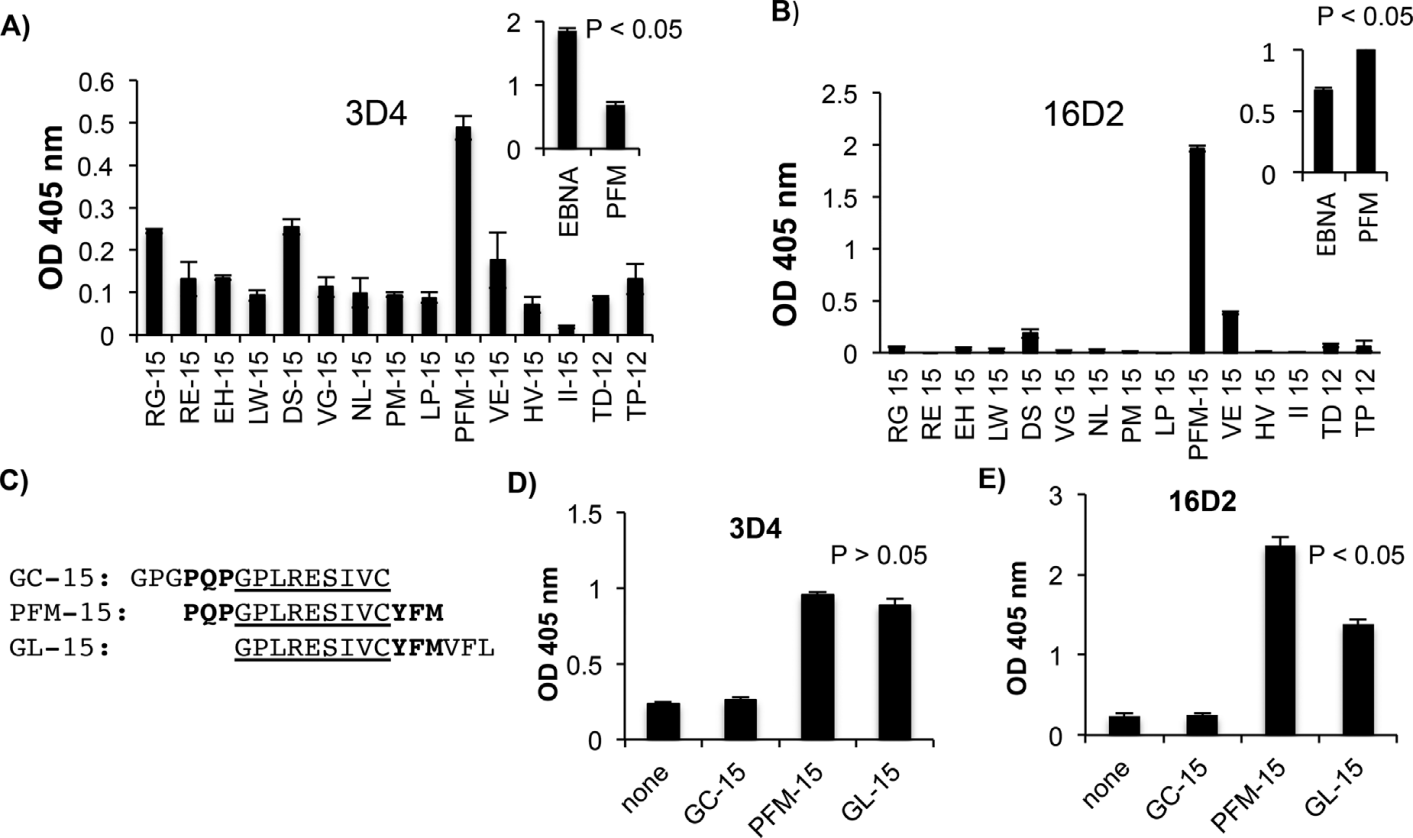
Peptide mapping. 3D4 (A) and 16D2 (B) were tested by ELISA for binding to peptides spanning the entire 148 amino acid VBS region. Insets compare binding of 3D4 and 16D2 to EBNA‐1 and PFM‐15. (C) Comparison of amino acid sequence of homologous peptides GC‐15, PFM‐15, and GL‐15. Common core region of all three peptides is underlined. Regions of overlap between GC‐15 and PFM‐15 and between PFM‐15 and GL‐15 are bolded. 3D4 (D) and 16D2 (E) were examined by ELISA for binding to GC‐15, GL‐15, and PFM‐15. 3D4 was used at a concentration of 5 μg/ml and 16D2 at 10 μg/ml for all above ELISAs. ELISAs are representative of two experiments. ELISA values were obtained in triplicate. Error bars represent standard deviations of triplicates. Standard t test (2 tail, type 1) performed to compare significance of binding to EBNA‐1 versus PFM in A and B and to compare significance of binding to PFM‐15 versus GL‐15 in D and E.

To more finely map the epitope/s recognized by 3D4 and 16D2, fifteen overlapping, linear oligopeptides spanning the entire 148 amino acid VBS domain in the carboxyl region of EBNA‐1 (EBNA_459‐607_) were synthesized and biotinylated at the amino terminal end (LifeTein, South Plainfield, NJ). All peptides were either 15 or 12 amino acids long with a 4–6 amino acid overlap (Table 3). Biotinylated peptides were attached to streptavidin coated ELISA plates and the binding of 3D4 and 16D2 to these peptides was examined. Both 3D4 and 16D2 bound stronger to peptide PFM‐15 than to any of the other overlapping peptides (Fig. 5A and B). However, 16D2 bound much more strongly to PFM‐15 than 3D4. When equimolar concentrations of B‐EBNA‐1 and B‐PFM‐15 were attached to streptavidin, 16D2 bound significantly more to B‐PFM‐15 than to B‐EBNA‐1 while 3D4 bound significantly less to B‐PFM‐15 than to B‐EBNA‐1 (Fig. 5A and B, insets). These results suggest that PFM‐15 is the optimal target of 16D2 but not of 3D4. 3D4 likely recognizes a conformational epitope that is partially comprised of PFM‐15. 3D4 displayed weak binding to several other peptides including RG‐15, (aa 459–473), DS‐15 (aa 499–513), and VE‐15 (aa 559–573). 16D2 also displayed weak binding to VE‐15. RG‐15 and DS‐15 show no homology to PFM‐15, however VE‐15 has some homology and shares a 5 amino acid overlap (VCYFM) at its 5′ end with the 3′termini of PFM‐15 (aa 549–563) (Table 3).

**Table 3:** Overlapping peptide sequences spanning the 148 amino acid VBS domain (EBNA_459‐607_) of EBNA‐1

Peptide	Amino acid sequence	Amino acid position
RG‐15	RKKGGWFGKHRGQGG	459–473
RE‐15	RGQGGSNPKFENIAE	469–483
EH‐15	ENIAEGLRALLARSH	479–493
LW‐15	LARSHVERTTDEGTW	489–503
DS‐15	DEGTWVAGVFVYGGS	499–513
VG‐15	VYGGSKTSLYNLRRG	509–523
NL‐15	NLRRGTALAIPQCRL	519–533
PM‐15	PQCRLTPLSRLPFGM	529–543
LP‐15	LPFGMAPGPGPQPGP	539–553
PFM‐15	PQPGPLRESIVCYFM	549–563
VE‐15	VCYFMVFLQTHIFAE	559–573
HV‐15	HIFAEVLKDAIKDLV	569–583
II‐15	IKDLVMTKPAPTCNI	579–593
TD‐12	TCNIRVTVCSFD	590–601
TP‐12	TVCSFDDGVDLP	596–607

Regions of amino amino acid overlap are underlined. Peptide recognized by 3D4 and 16D2 is shaded in gray.

The VBS has been crystallized in the laboratory of Dr. Lori Frappier in both the DNA bound and unbound forms 36, 37. Based on the crystal structure, we mapped peptide PFM‐15 to an exposed proline loop in the VBS domain at amino acids 549–563 37. The proline loop consists of a repeating P‐G motif followed by several 3′ terminal hydrophobic amino acids. To determine which amino acids in PFM‐15 are critical for the binding of 16D2 and 3D4, two additional overlapping peptides, GC‐15 (GPGPQPGPLRESIVC) (aa 546–560) and GL‐15 (GPLRESIVCYFMVFL) (aa 552–566) were synthesized and tested by ELISA for 3D4 and 16D2 binding (Fig. 5C, D, and E). GC‐15, GL‐15, and PFM‐15 have a common core of amino acids GPLRESIVC. GC‐15 shares three additional upstream amino acids (PQP) with PFM‐15, while GL‐15 shares 3 additional downstream amino acids (YFM) with PFM‐15 (Fig. 5C). Interestingly, neither 3D4 nor 16D2 bound GC‐15, yet both bound GL‐15, suggesting that amino acids YFM are critical for binding. 3D4 did not show a significant difference in binding GL‐15 compared to PFM‐15, while 16D2 displayed significantly higher binding to PFM‐15 than to GL‐15 (Fig. 5D and E). The only difference between these two peptides is that GL‐15 lacks PQP at its 5′ terminus and contains VFL at its 3′ terminus. PQP may be important for optimal binding of 16D2 while VFL may weaken it.

**Figure 6:**
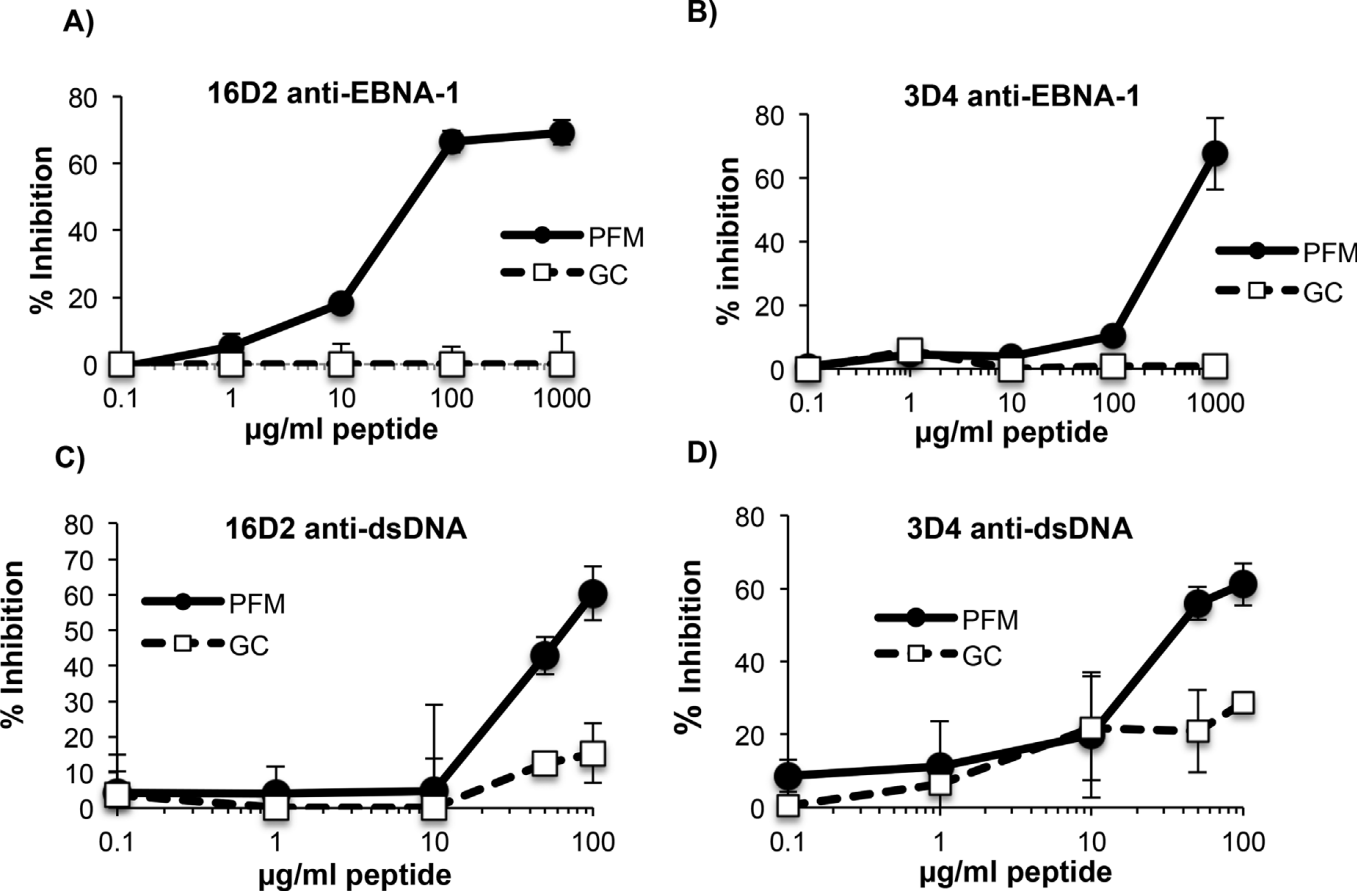
Peptide inhibition of MAbs binding to EBNA‐1 and dsDNA. 1D2 (5 μg/ml) and 3D4 (0.05 μg/ml) were incubated with increasing concentrations of peptides PFM‐15 or GC‐15 and examined by ELISA for binding to EBNA‐1 (A and B, respectively). 1D2 (5 μg/ml) and 3D4 (2.5 μg/ml) were incubated with increasing concentrations of peptides PFM‐15 or GC‐15 and examined by ELISA for binding dsDNA (C and D, respectively). ELISAs are representative of two experiments. ELISA values were obtained in triplicate. Error bars represent standard deviations of triplicate values.

We next examined whether PFM‐15 could competitively inhibit binding of 16D2 and 3D4 to EBNA‐1 (Fig. 6A and B) and dsDNA (Fig. 6C and D). A fixed concentration of 16D2 and 3D4 that resulted in 50% maximal binding to EBNA‐1 was incubated with increasing concentrations of peptide. At 100 μg/ml of peptide, PFM‐15 inhibited 16D2 from binding to EBNA‐1 by almost 70% (Fig. 6A) but inhibited 3D4 from binding to EBNA‐1 by only 10% (Fig. 6B). However, when the concentration of PFM‐15 was increased tenfold to1 mg/ml it also induced almost 70% inhibition of 3D4 binding to EBNA‐1. This inhibition was specific as 1 mg/ml of a control peptide, GC‐15 had no affect on 3D4 binding to EBNA‐1 (B). These results demonstrate that PFM‐15 can competitively inhibit binding of both antibodies to EBNA‐1 but that16D2 binds more strongly to PFM‐15 than 3D4. The observation that a high concentration of PFM‐15 is required to inhibit binding of 3D4 to EBNA‐1, further supports the hypothesis that this linear peptide is part of a conformational epitope that is optimally recognized by 3D4.

To examine whether PFM‐15 could inhibit binding to dsDNA, fixed concentrations of 16D2 and 3D4 that resulted in 50% maximal binding to dsDNA were incubated with increasing concentrations of peptide. At 100 μg/ml, PFM‐15 inhibited binding of both 16D2 and 3D4 to dsDNA by approximately 60% (Fig. 6C and D) suggesting that this peptide can mimic dsDNA and therefore partially block antibody binding to dsDNA.

### Characterization of four MAbs to EBNA‐1 that do not cross‐react with dsDNA

**Figure 7:**
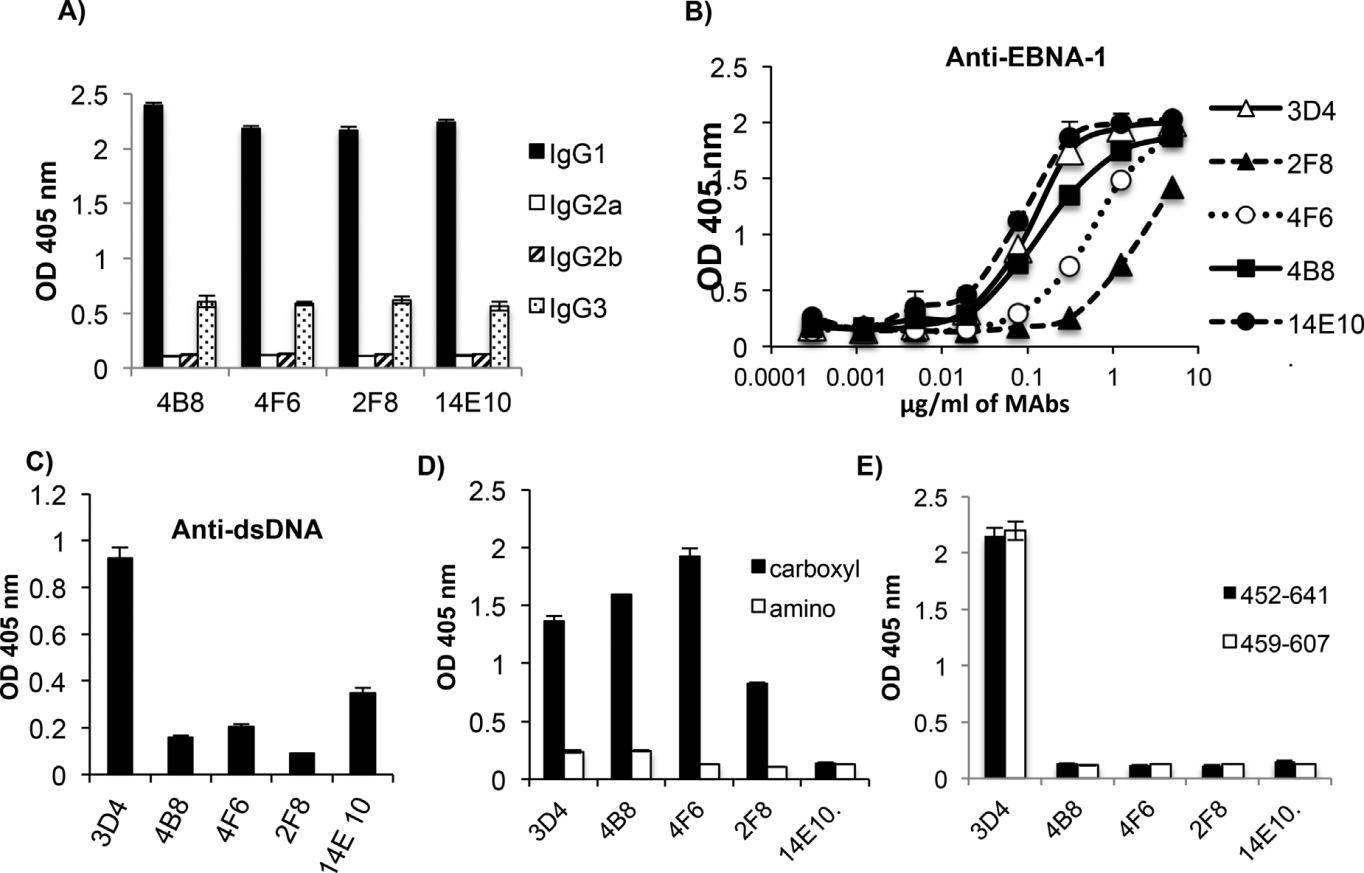
MAbs that bind to the carboxyl region of EBNA‐1 but do not cross‐react with dsDNA, recognize a different epitope than the cross‐reactive antibodies. (A) The IgG isotype of the non‐cross‐reactive MAbs 2F8, 14E10, 4F6, and 4B8 were determined by ELISA. (B) The non cross‐reactive MAbs, were tested by ELISA at a range of concentrations, for binding to EBNA‐1. 3D4 was used as the reference standard. MAbs were tested by ELISA for binding to dsDNA (C), to the amino and carboxyl regions of EBNA‐1 (D) and to two truncated carboxyl fragments; aa 452–641 and aa 459–607 (E). MAb concentrations used were 12.5 μg/ml in (C) and 1.25 μg/ml in (D) and (E). ELISAs are representative of two experiments. ELISA values were obtained in triplicate. Error bars represent the standard deviations of triplicates.

In the present study, we also generated four MAbs (14E10, 4B8, 4F6, and 2F8) to EBNA‐1 that do not cross‐react with dsDNA. We were interested in determining the region in EBNA‐1 that these antibodies bind relative to the cross‐reactive antibodies. The isotype used by all four of these antibodies was shown to be IgG1; the same isotype used by 3D4 (Fig. 7A). Binding to EBNA‐1 was examined at a range of concentrations, by ELISA and depicted in Figure 7B. 14E10 and 4B8 displayed similar binding to EBNA‐1 as 3D4, while 4F6 and 2F8 displayed weaker binding. Binding to dsDNA was also examined and depicted in Figure 7C. 4B8, 4F6, and 2F8 displayed negligible binding to dsDNA at a concentration of 12.5μg/ml, while 14E10 displayed a low but slightly higher level of binding to dsDNA than the other three MAbs (Fig. 7C). We next examined the binding of these MAbs at a concentration of 1.25 μg/ml to the amino and carboxyl regions of EBNA‐1. MAbs 4B8, 4F6, and 2F8 bound to the carboxyl region of EBNA‐1 but not the amino region (Fig. 7D). However, 14E10 failed to bind to either the amino or carboxyl regions (Fig. 7D) suggesting that it recognizes a conformational epitope in EBNA‐1 formed by the folding of the amino and carboxyl regions, or that it binds to an epitope that spans the junction of these two regions. We subsequently examined the binding of MAbs, 2F8, 4F6, and 4B8 to two of the truncated fragments (Fig. 3A and C) within the VBS in the carboxyl region of EBNA‐1; EBNA_452‐641_ and EBNA_459‐607_ (Fig. 7E). All three of these MAbs failed to bind to both truncated fragments although they recognized the complete carboxyl region (aa 410–641). Failure to bind to the larger of these two fragments (EBNA_452‐641_) suggests that amino acids upstream of the VBS, and 5′ of aa 452, are critical for recognition by these antibodies. It is interesting that only the cross‐reactive antibodies target an epitope that resides within the VBS.

## Discussion

In an earlier study, we identified an IgG antibody to EBNA‐1, 3D4, which cross‐reacts with dsDNA. In the present study we identified another antibody to EBNA‐1, 16D2, of the IgM isotype, which also cross‐reacts with dsDNA. We have compared the binding properties, sequences, and epitope specificity of both antibodies to determine if there are similarities that could help explain the structural properties that confer dual specificity.

Fine mapping of the target epitopes in EBNA‐1 for 3D4 and 16D2, revealed that they recognize an epitope localized to a 148 amino acid domain in the carboxyl region of EBNA‐1 that coincides with the VBS (aa 459–607). Epitope mapping using overlapping peptides that span the entire148 amino acid domain revealed that both 3D4 and 16D2 bind more strongly to peptide, PFM‐15 (PQPGPLRESIVCYFM) than any other linear peptide in this region. The binding of these MAbs to PFM‐15 is not due to charge interactions as PFM‐15 is hydrophobic and has a net charge of zero. Inhibition assays revealed that 10‐fold more PFM‐15 was required to inhibit 3D4 compared to 16D2 binding EBNA‐1. Furthermore, 3D4 bound equally well to PFM‐15 and the overlapping peptide, GL‐15, while 16D2 displayed significantly less binding to GL‐15 than PFM‐15 (Fig. 6). These results suggest that the epitopes targeted by the two antibodies share some homology but are not identical. The linear peptide PFM‐15 appears to be the optimal target epitope of 16D2 while 3D4 may prefer a structural epitope containing PFM‐15.

EBNA‐1 exists as a homodimer in latently infected cells. The VBS contains a core domain (aa 504–604) which mediates this dimerization 38. The VBS domain also binds viral dsDNA recognition sites in the EBV genome and it plays a role in the replication and mitotic segregation of the EBV episomes. Analysis of the crystallized structures of the VBS has enabled us to map the PFM‐15 peptide (aa 549–563) to an exposed proline loop in the core domain which projects from the EBNA‐1 dimer 36, 37. The proline loop has a proline rich tip (aa 545–549) and a 5′ arm (aa 538–544) that forms hydrogen bonds with the 3′ arm (aa 550–559), to stabilize the structure 37. PFM‐15 maps to the 3′ arm of this loop. Based on these known structures, we hypothesize that 3D4 optimally recognizes a conformational epitope that contains the linear sequence of PFM‐15 as well as non‐contiguous amino acids in the loop that form hydrogen bonds with amino acids in PFM‐15.

The observation that PFM‐15 could inhibit binding of both 3D4 and 16D2 to dsDNA suggests that this peptide can serve as a peptide mimetope for dsDNA at least at high molar concentrations. However, it is not yet clear from our amino acid sequence analyses and known structural data why 3D4 and 16D2 also recognize DNA and what aspects of PFM‐15 serve as the dsDNA mimic. The proline loop of EBNA‐1 has some structural flexibility and it is thought to undergo subtle structural changes when EBNA‐1 binds dsDNA 39. Therefore, there may be some structure in the loop in the region of the PFM‐15 peptide, which enables it to mimic DNA. It is interesting to note that the four MAbs that we examined in this study, which bind to EBNA‐1 but do not cross‐react with dsDNA, recognize an epitope that lies outside of the proline loop. We have also examined other peptide mimetopes of dsDNA described in the literature, to determine if there is any similarity to PFM‐15. In particular, we examined three peptides obtained from random phage display libraries, DWEYSVWLSN, ASPVTARVLWKASHV, and RLTSSLRYNP 22, 40, 41. There does not seem to be any sequence homology that can explain this shared property. Future studies will determine whether PFM‐15 alone can elicit an anti‐dsDNA response or whether it needs to be part of a proline loop structure to elicit that response.

The sequence analysis of the variable regions of 3D4 and 16D2 reveal differences and similarities that may help explain their shared dual binding properties. 3D4 and 16D2 utilize different V_H_ and V_L_ genes indicating that more than one anti‐EBNA‐1 antibody can cross‐react with dsDNA. 3D4 utilizes a gene from the V_H_7183 family and 16D2 utilizes a gene from the V_H_J558 family. Members of these two gene families are frequently observed among polyreactive antibodies that arise in the early immune response and among anti‐dsDNA autoantibodies 33, 42, 43, 44, 45. IgM antibodies that bind dsDNA are often germline encoded and are polyreactive. Although, the variable light chain gene of 16D2 is germline encoded, its variable heavy chain gene is mutated. It is unclear whether 16D2's autoreactivity is germline encoded or a consequence of somatic mutation. Future studies will address this by reverting the mutations in the CDR regions to germline and testing whether these antibodies still recognize dsDNA. 16D2 does not appear to be polyreactive, as it does not bind to LPS or the autoantigens Sm and PR3, frequently considered to be targets of polyreactive antibodies. However, it is unknown whether 16D2 is derived from a polyreactive precursor. Interestingly, both 3D4 and 16D2 use the same J_H_ and J_K_ regions and lack junctional diversity at the D‐J joint. They share a stretch of germline encoded amino acids at the carboxyl ends of their CDR3 regions. These may be important contact residues with the antigen. Moreover, both 3D4 and 16D2 contain arginine residues and phenylalanine and tyrosine residues in their V_H_ CDR3 regions and these basic and aromatic amino acids appear to be conserved in proteins that bind dsDNA 32, 33, 46. In addition, the CDR3 regions in the V_H_ of 3D4 and 16D2 are slightly longer than normal (33 and 39 amino acids, respectively), a characteristic of many autoantibodies 34, 35.

While EBNA‐1 has long been associated with SLE, most people who get infected with EBV do not develop lupus. This may be because healthy individuals who are exposed to EBV may preferentially develop antibodies to parts of EBNA‐1 that do not elicit cross‐reactivity, as observed with the non cross‐reactive MAbs that were generated in this study. Studies have shown that SLE patients have an altered humoral immune response to epitopes in EBNA‐1 relative to healthy individuals 47, 48, 49. SLE patients have been observed to preferentially have antibodies to epitopes in the amino and carboxyl regions of EBNA‐1 while healthy individuals have antibodies to the G‐A repeat region. The EBNA‐1 protein that we injected into mice in this study did not have a G‐A region, therefore the mice were forced to make an immune response to other regions of EBNA‐1. Antibodies to EBNA‐1 that cross‐react with dsDNA may be more likely to arise in genetically susceptible individuals who have particular MHC haplotypes.

It is interesting to speculate that other pathogens besides EBV may have proteins containing peptides homologous to PFM‐15 that can elicit anti‐dsDNA antibodies and therefore serve as a peptide mimetope of dsDNA. Protein BLAST was used to search for pathogen proteins containing peptides with homology to a stretch of amino acids in PFM‐15. Of particular interest were proteins that contain the YFM amino acid motif present at the 5′ terminus of PFM‐15. The presence of this motif appears to be critical for recognition by 3D4 and 16D2 since both MAbs failed to bind GC‐15, which shares a common peptide core with PFM‐15 but lacks YFM. The protein BLAST search identified a transporter protein derived from the bacteria Lachnospiraceae bacterium A2, which has 100% homology with seven contiguous amino acids in PFM‐15 (SIVCYFM) (see Table 3). Lachnospiracea belongs to the Clostridia class and is a part of the normal gut microbiota in mice and humans. A recent study demonstrated that young, female lupus prone mice have a significantly increased level of Lachnospiracea relative to age matched healthy controls 50. In addition, Lachnospiracea was observed to be overrepresented in female mice, which are associated with an earlier onset of SLE and more severe lupus like symptoms. These results suggest that gut microbiota may be a predisposing trigger for SLE and peptides derived from gut pathogens may lead to autoantibody production as a consequence of molecular mimicry.

In summary, this study describes an IgM MAb to EBNA‐1 that cross‐reacts with dsDNA and compares it to a previously identified IgG MAb that binds EBNA‐1 and cross‐reacts with dsDNA. Epitope mapping of EBNA‐1, using both these MAbs, identified a target peptide that lies at the base of the proline loop in the viral binding site of EBNA‐1. This peptide may serve as a mimetope for dsDNA. Future studies will determine whether this peptide can elicit an anti‐dsDNA response when injected into mice. If so, this will have important implications for treatment strategies aimed at preventing this peptide sequence from eliciting an autoimmune response following EBV infection or blocking cross‐reactive antibodies from binding to target autoantigens.

## Author Contributions

Pragya Yadav generated the 16D2 MAb and performed all of the initial anti‐dsDNA, anti‐EBNA‐1, and anti‐EBNA‐1 fragment ELISAs, performed the Crithidia assay, and generated the data for Figures 1–3. Matthew Carr did the PCR and the cloning of the heavy and light chain immunoglobulin genes for supplementary Figures 1–4. Ruby Yu performed the peptide ELISAs and generated most of the data for Figure 6. Alice Mumbey‐Wafula helped with all the MAb work and characterized the non cross‐reactive MAbs by ELISA and generated the data for Figure 5. Linda Spatz is the PI of the project who designed and supervised all of the experiments, generated the non‐cross‐reactive MAbs, performed peptide inhibition ELISAs, generated the data for Figures 4 and 7 and wrote the paper.

## Conflict of Interest

All authors of this manuscript concur that there is no conflict of interest; financial, commercial, or otherwise.

## Supporting information

Additional supporting information may be found in the online version of this article at the publisher's web‐site.

**Figure S1**. Variable heavy chain (V_H_) nucleic acid and amino acid sequences of 3D4 and comparison to most homologousV_H_ germline gene as determined by IgBLAST (NCBI database).
**Figure S2**. Variable light chain (V_L_) nucleic acid and amino acid sequences of 3D4 and comparison to most homologousV_L_ germline gene as determined by IgBLAST (NCBI database).
**Figure S3**. Variable heavy chain (V_H_) nucleic acid and amino acid sequences of 16D2 and comparison to most homologousV_H_ germline gene as determined by IgBLAST (NCBI database).
**Figure S4**. Variable heavy chain (V_L_) nucleic acid and amino acid sequences of 16D2 and comparison to most homologousV_L_ germline gene as determined by IgBLAST (NCBI database).
