# Supplementary material for: Mapping an epitope in EBNA‐1 that is recognized by monoclonal antibodies to EBNA‐1 that cross‐react with dsDNA
Source: Immun Inflamm Dis. 2016 Aug 2;4(3):362–75. doi: 10.1002/iid3.119 (PMC5004290; doi:10.1002/iid3.119)
Supplement: Supplementary file 1 — Figure S1. Variable heavy chain (VH) nucleic acid and amino acid sequences of 3D4 and comparison to most homologousVH germline gene as determined by IgBLAST (NCBI database). Figure S2. Variable light chain (VL) nucleic acid and amino acid sequences of 3D4 and comparison to most homologousVL germline gene as determined by IgBLAST (NCBI database). Figure S3. Variable heavy chain (VH) nucleic acid and amino acid sequences of 16D2 and comparison to most homologousVH germline gene as determined by IgBLAST (NCBI database). Figure S4. Variable heavy chain (VL) nucleic acid and amino acid sequences of 16D2 and comparison to most homologousVL germline gene as determined by IgBLAST (NCBI database). [file IID3-4-362-s001.pdf]

```
                ------------------------------------FR1-------------------------------------
                 L   Q   E   S   G   G   G   L   V   K   P   G   G   S   L   K   L   S   C   A
3D4HC:    10    CTG CAG GAG TCT GGG GGA GGC TTA GTG AAG CCT GGA GGG TCC CTG AAA CTC TCC TGT GCA  69
VH7183.14:      ... .GT ... ... ... ... ... ... ... ... ... ... ... ... ... ... ... ... ... ...
                 L   V   E   S   G   G   G   L   V   K   P   G   G   S   L   K   L   S   C   A

                ------- ---------CDR1------------- -------------------FR2---------------------
                 A   S   G   F   A   F   S   S   Y   D   M   S   W   V   R   Q   T   P   E   K
3D4HC:    70    GCC TCT GGA TTC GCT TTC AGT AGC TAT GAC ATG TCT TGG GTT CGC CAG ACT CCG GAG AAG  130
VH7183.14:      ... ... ... ... ... ... ... ... ... ... ... ... ... ... ... ... ... ... ... ...
                 A   S   G   F   A   F   S   S   Y   D   M   S   W   V   R   Q   T   P   E   K

                -------------------------- -------------CDR2-------------- ------------------
                 R   L   E   W   V   A   T   I   S   S   G   G   S   Y   I   Y   Y   P   D   S
3D4HC:    131   AGG CTG GAG TGG GTC GCA ACC ATT AGT AGT GGT GGT AGT TAC ATC TAC TAT CCA GAC AGT  190
VH7183.14:      ... ... ... ... ... ... ... ... ... ... ... ... ... ... .C. ... ... ... ... ...
                 R   L   E   W   V   A   T   I   S   S   G   G   S   Y   T   Y   Y   P   D   S

                ----------------------------------FR3---------------------------------------
                 V   K   G   R   F   T   I   S   R   D   N   A   R   N   T   L   Y   L   Q   M
3D4HC:    191   GTG AAG GGC CGA TTC ACC ATC TCC AGA GAC AAT GCC AGG AAC ACC CTG TAC CTG CAA ATG  250
VH7183.14:      ... ... ... ... ... ... ... ... ... ... ... ... ... ... ... ... ... ... ... ...
                 V   K   G   R   F   T   I   S   R   D   N   A   R   N   T   L   Y   L   Q   M

                ------------------------------------------------- ----------CDR3------------
                 S   S   L   R   S   E   D   T   A   L   Y   Y   C   A   R   P   P   P   F   Y
3D4HC:    251   AGC AGT CTG AGG TCT GAG GAC ACG GCC TTG TAT TAC TGT GCA AGA CCC CCC CCG TTC TAC  310
VH7183.14:      ... ... ... ... ... ... ... ... ... ... ... ... ... ... ...
                 S   S   L   R   S   E   D   T   A   L   Y   Y   C   A   R

                -------------- ----------------JH2----------------
                 F   D   Y   W   G   Q   G   T   T   L   T   V   S   S
3D4HC:    311   TTT GAC TAC TGG GGC CAA GGC ACC ACT CTC ACA GTC TCC TCA  352
```

SUPPLEMENTARY FIGURE 1

```
              -------------------------------------FR1------------------------------------
              T   Q   S   P   L   S   L   P   V   S   L   G   E   Q   A   S   I   S   C   R
3D4LC:   13  ACC CAA TCT CCA CTC TCC CTG CCT GTC AGT CTT GGA GAG CAA GCC TCC ATC TCT TGC AGA  72
bb1:         ... ... A.. ... ... ... ... ... ... ... ... ... ..T ... ... ... ... ... ... ...
              T   Q   T   P   L   S   L   P   V   S   L   G   D   Q   A   S   I   S   C   R

             ------  ------------------CDR1-----------------  ------------FR2-----------
              S   S   Q   S   L   V   H   S   N   G   N   T   Y   L   H   W   Y   L   Q   K
3D4LC:   73  TCT AGT CAG AGC CTT GTA CAC AGT AAT GGA AAC ACC TAT TTA CAT TGG TAC CTG CAG AAG  132
bb1:         ... ... ... ... ... ... ... ... ... ... ... ... ... ... ... ... ... ... ... ...
              S   S   Q   S   L   V   H   S   N   G   N   T   Y   L   H   W   Y   L   Q   K

             -------------------------------------  ---CDR2---  ---------FR3-------------
              P   G   Q   S   P   K   L   L   I   Y   K   V   S   N   R   F   S   G   V   P
3D4LC:  133  CCA GGC CAG TCT CCA AAG CTC CTG ATC TAC AAA GTT TCC AAC CGA TTT TCT GGG GTC CCA  192
bb1:         ... ... ... ... ... ... ... ... ... ... ... ... ... ... ... ... ... ... ... ...

             ------------------------------------------------------------------------------
              D   R   F   S   G   S   G   S   G   T   D   F   T   L   K   I   S   R   L   E
3D4LC:  193  GAC AGG TTC AGT GGC AGT GGA TCA GGG ACA GAT TTC ACA CTC AAG ATC AGC AGA CTG GAG  252
bb1:         ... ... ... ... ... ... ... ... ... ... ... ... ... ... ... ... ... ... G.. ...
              D   R   F   S   G   S   G   S   G   T   D   F   T   L   K   I   S   R   V   E

             --------------------------------  ----------------CDR3---------------  ---
              A   E   D   L   G   F   Y   F   C   S   Q   T   T   H   V   P   W   T   F   G
3D4LC:  253  GCT GAG GAT CTG GGA TTT TAT TTC TGC TCT CAA ACT ACA CAT GTT CCG TGG ACG TTC GGT  312
bb1:         ... ... ... ... ... G.. ... ... ... ... ... .G. ... ... ... ...
              A   E   D   L   G   V   Y   F   C   S   Q   S   T   H   V   P

             -------Jk1------
              G   G   T   K
3D4LC:  313  GGA GGC ACC AAG  324
```

SUPPLEMENTARY FIGURE 2

```
                 -----------------------------FR1---------------------------------
                 Q   L   Q   E   S   G   A   E   L   V   R   P   G   T   S   V   K   I   S   C
16D2HC:   7      CAG CTG CAG GAG TCT GGA GCT GAG CTG GTA AGG CCT GGG ACT TCA GTG AAG ATA TCC TGC  67
J558.16:         ... T.. ... C.. ... ... ... ... ... ... ... ... ... ... ... ... ... ... ... ...
                 Q   L   Q   Q   S   G   A   E   L   V   R   P   G   T   S   V   K   I   S   C

                 ---------  -------------CDR1----------  ---------------FR2------------
                 K   A   S   G   Y   A   F   T   N   Y   W   L   G   W   V   K   Q   R   P   G
16D2HC:   68     AAG GCT TCT GGA TAC GCC TTC ACT AAC TAC TGG CTA GGT TGG GTA AAG CAG AGG CCT GGA  127
J558.16:         ... ... ... ..T ... A.. ... ... ... ... ... ... ... ... ... ... ... ... ... ...
                 K   A   S   G   Y   T   F   T   N   Y   W   L   G   W   V   K   Q   R   P   G

                 ----------------------------  -------------CDR2-------------   -------------
                 H   G   L   E   W   I   G   D   I   Y   P   G   S   G   N   T   Y   Y   N   E
16D2HC:   128    CAT GGA CTT GAG TGG ATT GGA GAT ATT TAC CCT GGA AGT GGT AAT ACT TAC TAC AAT GAG  187
J558.16:         ... ... ... ... ... ... ... ... ... ... ... ... G.. ... T.. ... A.. ... ... ...
                 H   G   L   E   W   I   G   D   I   Y   P   G   G   G   Y   T   N   Y   N   E

                 -----------------------------------FR3-------------------------------------
                 K   F   K   G   K   A   T   L   T   A   D   K   S   S   S   T   A   Y   M   Q
16D2HC:   188    AAG TTC AAG GGC AAA GCC ACA CTG ACT GCA GAC AAA TCC TCG AGC ACA GCC TAT ATG CAG  247
J558.16:         ... ... ... ... ..G ... ... ... ... ... ... .C. ... ..C ... ..T ... ..C ... ...
                 K   F   K   G   K   A   T   L   T   A   D   T   S   S   S   T   A   Y   M   Q

                 ----------------------------------------------------  ---------CDR3---------
                 L   S   S   L   T   S   E   D   S   A   V   Y   F   C   A   R   I   L   R   L
16D2HC:   248    CTC AGT AGC CTG ACA TCT GAG GAC TCT GCT GTC TAT TTC TGT GCA AGG ATA CTA CGG CTA  307
J558.16:         ... ... ... ... ... ... ... ... ... ... ... ... ... ... ...
                 L   S   S   L   T   S   E   D   S   A   V   Y   F   C   A

                 -------------------------  ---------JH2----------
                 R   Q   Y   F   D   Y   W   G   Q   G   T   T   L
16D2HC:   308    CGT CAG TAC TTT GAC TAC TGG GGC CAA GGC ACC ACT CTC  346
J558.16:
```

SUPPLEMENTARY FIGURE 3

```
                 -------------------------------------FR1-----------------------------------
                  T   Q   S   P   S   S   L   A   V   S   V   G   E   K   V   T   M   S   C   K
16D2LC:     13   ACC CAG TCT CCA TCC TCC CTA GCT GTG TCA GTT GGA GAG AAG GTT ACT ATG AGC TGC AAG   72
8-30:            --- ... ... ... ... ... ... ... ... ... ... ... ... ... ... ... ... ... ... ...
                      Q   S   P   S   S   L   A   V   S   V   G   E   K   V   T   M   S   C   K

                 ------- -------------------CDR1--------------------- ----------FR2----------
                  S   S   Q   S   L   L   Y   S   S   N   Q   K   N   Y   L   A   W   Y   Q   Q
16D2LC:     73   TCC AGT CAG AGC CTT TTA TAT AGT AGC AAT CAA AAG AAC TAC TTG GCC TGG TAC CAG CAG   132
8-30:            ... ... ... ... ... ... ... ... ... ... ... ... ... ... ... ... ... ... ... ...
                  S   S   Q   S   L   L   Y   S   S   N   Q   K   N   Y   L   A   W   Y   Q   Q

                 ----------------------------------------- ----CDR2--- ----------------------
                  K   P   G   Q   S   P   K   L   L   I   Y   W   A   S   T   R   E   S   G   V
16D2LC:    133   AAA CCA GGG CAG TCT CCT AAA CTG CTG ATT TAC TGG GCA TCC ACT AGG GAA TCT GGG GTC   192
8-30:            ... ... ... ... ... ... ... ... ... ... ... ... ... ... ... ... ... ... ... ...
                  K   P   G   Q   S   P   K   L   L   I   Y   W   A   S   T   R   E   S   G   V

                 --------------------------------------FR3----------------------------------
                  P   D   R   F   T   G   S   G   S   G   T   D   F   T   L   T   I   S   S   V
16D2LC:    193   CCT GAT CGC TTC ACA GGC AGT GGA TCT GGG ACA GAT TTC ACT CTC ACC ATC AGC AGT GTG   252
8-30:            ... ... ... ... ... ... ... ... ... ... ... ... ... ... ... ... ... ... ... ...

                 ------------------------------------- ---------------CDR3------------------
                  K   A   E   D   L   A   V   Y   Y   C   Q   Q   Y   Y   S   Y   P   W   T   F
16D2LC:    253   AAG GCT GAA GAC CTG GCA GTT TAT TAC TGT CAG CAA TAT TAT AGC TAT CCG TGG ACG TTC   313
8-30:            ... ... ... ... ... ... ... ... ... ... ... ... ... ... ... ... ...
                  K   A   E   D   L   A   V   Y   Y   C   Q   Q   Y   Y   S   Y   P

                 ---------Jk1--------
                  G   G   G   T   K
16D2LC:    314   GGT GGA GGC ACC AAG   318
```

SUPPLEMENTARY FIGURE 4

SUPPLEMENTARY FIGURE LEGEND

Supplementary Figure 1. Variable heavy chain (V_H) nucleic acid and amino acid sequences of 3D4 and comparison to most homologous V_H germline gene as determined by IgBLAST (NCBI database).

Supplementary Figure 2. Variable light chain (V_L) nucleic acid and amino acid sequences of 3D4 and comparison to most homologous V_L germline gene as determined by IgBLAST (NCBI database).

Supplementary Figure 3. Variable heavy chain (V_H) nucleic acid and amino acid sequences of 16D2 and comparison to most homologous V_H germline gene as determined by IgBLAST (NCBI database).

Supplementary Figure 4. Variable heavy chain (V_L) nucleic acid and amino acid sequences of 16D2 and comparison to most homologous V_L germline gene as determined by IgBLAST (NCBI database).
